# Recruitment, training and supervision of nurses and nurse assistants for a task-shifting depression intervention in two RCTs in Brazil and Peru

**DOI:** 10.1186/s12960-021-00556-5

**Published:** 2021-02-05

**Authors:** Thais Izabel Ugeda Rocha, Suzana Crismanis de Almeida Lopes Aschar, Liliana Hidalgo-Padilla, Kate Daley, Heloísa Garcia Claro, Hellen Carolina Martins Castro, Daniela Vera Cruz dos Santos, J. Jaime Miranda, Ricardo Araya, Paulo Rossi Menezes

**Affiliations:** 1 Faculty of Medicine of Sao Paulo University-Preventive Medicine Department, Av. Dr. Arnaldo, 455-Cerqueira César, sala 2364, Sao Paulo, SP 01246-903 Brazil; 2 CRONICAS Center of Excellence in Chronic Diseases, Universidad Peruana Cayetano Heredia, Av. Armendáriz 445, Miraflores, Lima, Peru; 3 Centre for Global Mental Health and Primary Care Research, Health Service and Population Research, Institute of Psychiatry, Psychology and Neuroscience, King’s College London, 18 De Crespigny Park, London, SE5 8AF UK

**Keywords:** Task-shifting, mhealth, Mental health, Recruitment, Supervision, Training, Depression, Diabetes, Hypertension

## Abstract

**Background:**

Task-shifting and technology in psychological interventions are two solutions to increasing access to mental health intervention and overcoming the treatment gap in low and middle-income countries. The CONEMO intervention combines a smartphone app with support from non-specialized professionals, aiming to treat depression in patients with diabetes and/or hypertension. The aim of this paper is to describe the process of recruitment, training and supervision of the non-specialized professionals who participated in the CONEMO task-shifting intervention in Brazil and Peru.

**Methods:**

We described and analyzed data related to the recruitment, training and supervision of 62 nurse assistants from the health system in Sao Paulo, Brazil, and three hired nurses in Lima, Peru. The data were collected from information provided by nurses and nurse assistants, supervisor records from supervision meetings and the CONEMO platform database.

**Results:**

We found that task-shifting was feasible using existing resources in Sao Paulo and additional human resources in Lima. Training and supervision were found to be crucial and well received by the staff; however, time was a limitation when using existing human resources. Ensuring technological competence prior to the start of the intervention was essential. Group supervision meetings allowed non-specialized professionals to learn from each other’s experiences.

**Conclusion:**

Carefully considering recruitment, training and supervision of non-specialized professionals is important for effective task-shifting when delivering an mHealth intervention for depression. Opportunities and challenges of working in different health systems are described, which should be considered in future implementation, either for research or real settings.

*Trial registration* NCT028406662 (Sao Paulo), NCT03026426 (Peru).

## Background

Mental health has significant importance to public health worldwide. In low and middle-income countries (LMICs), mental disorders account for 11.1% of the total burden of disease [1–3], yet only 10–25% of people with mental health conditions receive treatment [3–5]. One contributory factor to this treatment gap is the low number of mental health workers in LMICs: it is estimated an additional 1.18 million mental health professionals are required to attend basic needs [6]. As a result, mental health problems go unrecognized and untreated, leading to chronicity, psychological suffering and long-term elevated costs of care [3–8]. Effective strategies are needed to overcome this gap. In Brazil and Peru, there have been efforts to implement mental health reforms to increase access; however, the lack of financial and human resources are significant barriers [9].

Task-shifting was proposed by the World Health Organization (WHO) as one possible solution. This is to redistribute tasks to non-specialized health workers, enabling more efficient use of resources [10]. Digital technology could facilitate task-shifting. For example, using smartphone applications to deliver psychoeducational material directly to patients and standardizing specific tasks to reduce the need for specialized human resources. In 2017, the World Bank estimated that there was one mobile cellular subscription per person in LMICs, estimates in Brazil and Peru were even higher [11]. The diffusion of smartphones and internet access in LMICs makes this solution feasible.

There are some challenges in implementing task-shifting which must be addressed to maximize success, this includes inadequate training of non-specialized health workers and a lack of supervision by competent health care professionals [12]. To facilitate a rapid increase in workforce capacity, task-shifting would require competency-based training to prepare workers to perform delineated tasks [13]. This would be quicker and more cost-effective than training specialized professionals [14]. Supervision would also be required for safety.

The Latin America Treatment Innovation Network in Mental Health (LATIN-MH) is one of five hubs funded by the US-National Institute of Mental Health (NIMH) to research the use of task-shifting in LMICs to address mental health problems. LATIN-MH conducted two Randomized Controlled Trials (RCTs) to evaluate a task-shifting intervention, one in Brazil, one in Peru. Participants were being treated for diabetes and/or hypertension in the public health system and were experiencing depressive symptoms. The intervention (CONEMO) aims to reduce depressive symptoms that often coexist with chronic health conditions. CONEMO consists of 18 sessions, delivered three times a week, over a course of 6 weeks. It is based on Behavior Activation, delivered by smartphones and supported by nurses or nurse assistants^1^ (NA). Nurses/NA trained participants to use CONEMO, monitored app usage and adherence, called participants to follow-up on progress or encourage adherence; and gave technical support when necessary. The CONEMO platform has two interfaces: a participant-facing smartphone application and a professional-facing dashboard used to monitor participant progress. The platform assists in the supervision and enables data gathering. Given the known challenges in implementing task-shifting, nurses/NA were given training and supervision throughout.

### Aim

The aim of this paper is to provide a description of the process of recruitment, training and supervision of non-specialized workers (nurse/NAs) who participated in a task-shifting intervention in two RCTs in Sao Paulo and Lima.

## Methods

### Study setting/design

In Sao Paulo, the study took place in 20 public primary care centers, known as family health units (FHUs). Primary care is the main gateway to the health system in Brazil [15] and FHUs are divided into teams that typically comprise at least one General Practitioner, nurse, NA and Community Health Worker [16]. Each FHU team covers an area with an average of 3000 inhabitants [15]. The nursing science in Brazil is practiced by nurses (undergraduate degree), nurse technicians (technician degree) and nurse assistants (capacitation degree), differing in their level of skills and responsibilities. Each FHU team had two NAs, only one of whom was included in the research. NAs were employed for 40 h per week and their typical tasks included vaccinations, changing bandages, and administering medication. Although some NAs were overwhelmed by their workload, due to the number of NAs and organizational structure, a decision was made to use existing human resources. In the trial they were required to complete the CONEMO activities alongside these tasks.

In Lima, the study was conducted in primary health care centers from the social security network and outpatient services from three hospitals from the Ministry of Health network. A pilot study found nurses in Peru were overwhelmed with the number of tasks assigned to them, and, with a few exceptions, were not motivated to invest time in additional responsibilities [17]. This was a likely barrier to implementation so the research team decided to hire full-time nurses to conduct the intervention. The nurses were employed for 48 h per week and worked full-time with CONEMO activities for the duration of the trial.

RCT enrollment started in September 2016 in Sao Paulo, and in January 2017 in Lima. Data collection was completed in April 2018.

### Nurses/NAs recruitment

In Sao Paulo, the FHU Manager selected the NAs to participate, they were asked to prioritize those motivated to participate and preferably skilled in operating electronic devices. No prior contact or specialization in mental health was required and the aim was to recruit 52 NAs, one from each FHU team.

In Lima, the research team made a public call for licensed nurses who were technologically literate and had at least 1 year of experience working in health centers. Experience of patients with depression or chronic diseases was desirable, but not mandatory. Candidates were interviewed and participated in a role-play to assess the capacity to work with electronic devices; three nurses were recruited.

### Procedures

#### Training

Following recruitment, nurses/NAs received training consisting of three components. The first was theoretical where nurses/NAs received information regarding the study, the intervention, and tasks to complete. The second was practical, where they had the chance to practice initial appointments and telephone calls with the research team or colleagues. The third was ethical training covering good clinical practice in research; this was considered essential as nurses/NAs do not all complete this as part of their core professional training. Also, a set of manuals was provided.

In Sao Paulo, an 8-h training was delivered by the clinical research team (two Psychologists and a Research Assistant) at a location provided by the organization. NAs were organized into four groups, each group attending one session to minimize the impact on the FHU routines. In Lima, a 40-h training was conducted by the Clinical Coordinator and the Trial Manager, both Psychologists. As nurses were working full-time on CONEMO, they were able to have a longer and more detailed training program.

At the end of the training, nurses/NA completed an 18-item questionnaire to evaluate their knowledge before starting to work with trial participants. Both cities used the same evaluation and scoring guidelines, and in cases where a nurse/NA scored less than 75%, additional training was given. This was tailored to the individual and the identified knowledge gaps.

#### Supervision

The trial had three clinical supervisors (two in Sao Paulo, one in Lima) responsible for supervising nurses/NAs in task-shifting. Clinical supervisors conducted weekly meetings, in groups or individually, by phone or in person, according to need and availability. The aim was to discuss tasks, resolve difficulties, and answer questions on depressive symptoms, ethics or practical aspects of CONEMO. This also aimed to ensure fidelity to the trial protocol. The supervisor dashboard enabled supervisors to see participant progress and nurses/NA adherence to tasks. It also alerted supervisors to help requests made by nurses outside of supervision.

#### Measures and data collection

Nurses/NAs provided information on the number of years they had worked at the FHU (in Sao Paulo) and their educational level and graduation year. The number of training hours completed by each nurse/NA was also recorded.

Data collected from the CONEMO platform included the number of participants assigned to each nurse/NA, number of scheduled activities, completion of calls and appointments and time spent working on programmed tasks (initial and final appointment and follow-up calls). Clinical supervisors kept records from each supervision meeting, they recorded supervision duration, modality (individual or group) and subjects discussed. Subjects were selected from a list of 17 possible topics: serious adverse events, canceled tasks, difficult situations related to the study, treatment termination, resolved help request, unresolved help request, administrative issues, additional contacts, participants’ difficulties with CONEMO, non-adherence, questions regarding tasks, non-connectivity call, delayed tasks/nurse/NA adherence, difficulties to contact the participant, other scheduled contact points, initial appointment and others. They also recorded any additional issues and reasons for absence.

### Data handling and analysis

Data were exported from the CONEMO platform to a CSV file and the database was password protected. Information from supervisor records was extracted and organized in a spreadsheet format. Descriptive statistics and frequencies were calculated. Variables analyzed were sample characteristics such as gender, professional background, experience; and training and supervision variables such as training scores, attendance rate, number of participants per nurse/NAs, the status of tasks (overdue and on time) and help requests. No missing data were identified.

## Results

### Recruitment and sample characteristics

In Sao Paulo, FHU Managers were requested to conduct the recruitment; however, in reality, this was mostly completed by the head nurse since they had more knowledge about NAs’ competences. Of the 52 NAs initially recruited, seven left before the trial started. The reasons for leaving were medical leave, transfer to another health facility, or declining to participate due to not feeling confident working with technology or for being already overwhelmed with existing tasks. They were replaced and their data excluded. Of those who started the trial, three dropped out for similar reasons. They were replaced but as they participated for a significant period of time, their data were included. In total 62 professionals were trained and 55 participated. Of those participating, 38 (69%) were nurse assistants, 17 (31%) were nurse technicians. Although their academic degree is different, their role in the health system is the same. The majority were female (95%). In terms of experience, 24 (44%) had between 1 and 5 years of experience in primary health care, 17 (31%) 6–10 years, and 14 (25%) had more than 10 years of experience. They all had little or no knowledge of mental health. NAs monitored an average of eight participants, with a minimum of two and a maximum of 15, in addition to their regular FHU activities.

In Lima, eight candidates applied, six met selection criteria and three were recruited. All were female and had an average of 3 years of experience within the healthcare system. All reported little knowledge of mental health. Each nurse monitored between 72 and 74 participants and worked full-time on CONEMO.

### Training

In Sao Paulo, all NAs completed an 8-h standard training course and three (5%) required additional training because they scored below 75% on the training evaluation. The additional training covered the CONEMO app, dashboard functions and the intervention itself. In Lima, all nurses completed a 40-h training course and all scored over 75% on the test; therefore, no additional training was required.

### Supervision

In Sao Paulo, 620 supervision meetings between 2 supervisors and 55 NAs were completed over a 15-month period. These comprised 373 (60%) individual meetings (103 h, 32%), 240 (39%) group supervisions (212 h, 67%), and seven meetings completed by telephone (1 h, 1%). Meetings were held weekly in each facility and their length varied according to needs. On average each supervisor held 5 meetings per week, the average duration of an individual meeting was 16 min, and 53 min for group meetings. NAs received an average of 5 h and 45 min of supervision, approximately 43 min per participant.

In Lima, 88 supervision meetings between the supervisor and the three nurses were completed over a 10-month period. These comprised 47 (53%) individual meetings (19 h, 29%), 34 (39%) group meetings (47 h, 70%) and 7 (8%) individual meetings completed by telephone (1 h, 1%). Meetings were held weekly in the research team’s office. On average, each nurse attended one supervision meeting per week, and the supervisor held two meetings per week. The average duration of individual meetings was 24 min, and 83 min for group meetings. On average each nurse received 22 h of supervision which translates to 18 min per participant.

Adherence to supervision meetings and reasons for absence were explored. In Sao Paulo, there were a total of 289 absences, 5.5 per NA on average. The most common reason for not attending was vacation, with 120 missed meetings (42%). 72 meetings were missed for medical leave (25%), 65 for being unable to leave FHU regular activities (22%), and 32 were missed without previous notice or further explanation (11%). In Lima, there was only one absence which was due to medical leave.

In both sites, supervision meetings addressed questions related to the intervention and follow-up, including activities and tasks carried out during the week. The most frequently discussed subjects in Sao Paulo were: initial appointment (17.5%), other scheduled contact points (15.6%) and difficulties contacting the participant (15.5%). In Lima, the most frequently addressed topics were: Other scheduled contact points (13.5%), non-connectivity calls (11.8%) and initial appointment (11.5%). The number of help requests from NA/nurses to supervisors outside of supervision meetings was 12 in Sao Paulo and one in Lima. Of all the tasks completed by NA/nurses, 2472 were completed on time in Sao Paulo (84%), and 1764 (97%) in Lima.

## Discussion

This paper allows reflection on the process of recruiting, training and supervising non-specialized health workers to deliver technology-based task-shifting interventions which have been recognized as an important research gap [18]. The knowledge acquired with these experiences can be a great asset to implementation planning and future research.

### Recruitment

In Brazil, primary care was previously composed of personnel with no formal training, but in a journey of healthcare improvement, mandatory qualifications were introduced increasing the number of NAs available [19]. In Sao Paulo, this opened up the possibility of task-shifting using existing NAs who play a key role in primary care services. In Lima, nurses usually deliver health programs targeting specific populations, and so they were chosen under the assumption that if CONEMO was effective and not too onerous, it could be included as an additional program, making the intervention sustainable [17]. However, as existing nurses reported being overburdened, additional nurses were hired for the purpose of the trial. Both approaches were successful in terms of tasks completion and supervision attendance, which highlighted that local service context and available resources should steer recruitment.

In terms of experience, a significant proportion of NAs in Sao Paulo had worked in the FHU region for a long time. This meant they were closer to the participants than other health professionals and were aware of their medical and personal histories. It was hypothesized that this could facilitate the intervention. However, as NAs with less experience in the region and Lima nurses who were not based in a particular health center prior to the intervention also performed tasks adequately, this experience was not essential.

A previous lack of knowledge or experience dealing with mental health issues did not appear to be a barrier to implementation. In Sao Paulo, some inaccurate beliefs about depression were identified, such as understanding it as deep sadness or lack of willpower, but could be corrected through the training and supervision provided. In Lima, even though nurses knew their tasks were not aimed at providing psychological support, they requested a brief talk about depression. Technical challenges were however flagged in Sao Paulo as an issue throughout, which highlighted the need to ensure technological literacy and support.

In Sao Paulo, a number of NAs dropped out due to logistical or personal reasons. This could have been minimized by service planning and addressing motivation to participate. Conversely, Lima did not experience nurse attrition, likely due to nurses being recruited and hired specifically for the role. Motivation and setting realistic expectations would be important to consider when planning recruitment to ensure sustainability.

### Training

The duration of training differed between sites. In Sao Paulo, NAs could not be absent from the FHU for more than 1 day, since it would imply delays in their work schedule, so the training content was compressed into a shorter period of time. In Lima, the training content was delivered over a longer time frame, since they were hired full-time for this work. This enabled nurses to have more time to practice, understand procedures and gain confidence. This was evidenced by all nurses passing the training evaluation. A small number of NAs in Sao Paulo did not pass and required additional training, highlighting the importance of evaluating learning and ensuring training packages are comprehensive and allow time for assimilation in order to guarantee the delivery of the intervention with adequate fidelity. It may, of course, be that in Lima a shorter training package could deliver the same results.

### Supervision

Supervision was also shown to be essential for task-shifting. Through supervision, important questions were raised, nurses/NAs improved knowledge and skills, and maintained fidelity to the intervention. Supervision was well received by nurse/NAs. In Sao Paulo, there was a significant number of missed meetings, but the majority (89%) were for reasons beyond the NAs control such as the FHU busy routine or unplanned medical leave. In Lima, nurses did not have tasks outside of CONEMO and so they were able to prioritize supervision meetings. However, as appointments were scheduled around participant availability, supervision had to be rescheduled at times. Boundaried time to attend supervision and flexibility in the supervisors’ schedule or location would be useful.

Individual supervision meetings were more frequent than group supervision in Sao Paulo, although naturally shorter in duration. However, when we consider the number of NAs and the variant interval between meetings, they required 3.3 times more supervisor time. In some instances, supervisors in Sao Paulo had to attend the FHU more than once a week (e.g., if NAs missed too many meetings in a row); therefore, they needed to be available for more time than predicted. In Lima, group supervision meetings were longer due to the number of participants each nurse was responsible for. Even though individual meetings were more frequent, they generally took place in addition to the group meetings and were performed in specific cases, especially at the beginning of the intervention to address questions regarding the intervention. Unsurprisingly, NAs with less experience with technology required more supervision support, highlighting the need for competence in this area. Given the issues discussed and caseload of each nurse/NA, weekly meetings appeared to be a reasonable frequency. When planning future implementation, the format of supervision should be based on which is more cost-effective for the health system and the best fit for the service context. Remote supervision could be a way to reduce the time required by the supervisor.

The issues brought to supervision were similar across sites over time. According to the intervention manual, it was mandatory to discuss all initial appointments. Nurses/NAs shared their experiences and challenges when training participants to use the app. Group supervision meetings were helpful in this matter, as the staff contributed with possible solutions to the issues raised. Difficulties contacting participants were a frequent subject in both sites, often due to the participant working, traveling, or providing inaccurate contact details. In Lima, lack of connectivity was frequently discussed, mainly where participants accidentally closed the app or ran out of battery. Some of these practical issues could be overcome by writing solutions into the manual and avoiding the need for supervision on these matters.

Nurses/NAs could contact the supervisor through the CONEMO system outside of supervision. This was predominately used for technological issues and never for a mental health emergency. Whilst helpful to have this safety net, it is unlikely to require heavy supervisor resources going forward. Communication between nurse/NAs and supervisors took place more frequently through an instant messaging app than on the CONEMO platform as this proved an easier and faster way to communicate. This flagged the need to utilize existing and easily accessed systems when possible, but caution is required to ensure the confidentiality of participants and security of data.

Supervision meetings in Sao Paulo were often used for the completion of tasks, similar to the experience in the pilot studies [17, 20]. This was due to NAs struggling to find time in the week to dedicate to CONEMO and so they used this time to get on top of tasks, which explains the average supervision time spent for each participant being 2.4 times higher in Sao Paulo than in Lima. This learning demonstrated the need for boundaried and realistic time frames to accomplish tasks when they are part of an existing job role, and the usefulness of a mechanism to monitor and track task completion.

In terms of capacity, supervision appeared to demand more time at the beginning of the implementation, as nurses/NAs were familiarizing themselves with the CONEMO platform and the specific issues participants presented with. Over time, they learned how to manage some difficulties and required less help from the supervisor. In that sense, the human resource management requirement would be likely to reduce over time if the staff is retained, which would increase both supervisors and nurses/NA’s work capacity.

CONEMO was able to reach significantly more people than three psychologists, such as the supervisors, could have reached on their own within the same time period (657 participants in both sites in approximately 1.5 years). Considering there are 12.4 and 9.5 psychologists per 100,000 inhabitants in Brazil and Peru [21, 22], respectively, interventions like CONEMO can help reduce the burden. Even though implementing task-shifting will not cover the high needs for mental health coverage [23], this study shows that nurses/NAs can support low-level psychological interventions. Through taking on a supervisory role in task-shifting, psychologists could facilitate access to mental health interventions for a greater proportion of the population and free up specialist resources to treat more complex cases.

## Limitations

One limitation of the study was that it relied on self-report data from nurses/NAs and supervisors regarding the length of time it took to complete a task. With supervisors, only time spent in supervision was recorded, which underestimates the supervisor time required for transportation and administration related to CONEMO. Additionally, the unplanned use of instant messaging tools to communicate with nurse/NAs, led to data not being fully captured on the CONEMO platform, which led to some underestimates and missing data regarding self-requests.

Another limitation is that, although the intervention and platforms used were the same in both sites, the settings involved were different. Therefore, it is not possible to compare sites and draw any clear conclusions regarding working with an existing system or hiring additional staff, or the optimum training and supervision required. It is likely there is not a one size fits all, and implementation must be adapted for the health system.

## Conclusion

This paper describes the recruitment, training and supervision of nurse/NAs implementing a task-shifting intervention, using a smartphone application to treat depression in Sao Paulo and Lima. The study showed that it is feasible to successfully implement an intervention with less specialized staff within the public health system, but that doing so requires appropriate and carefully considered adaptation to the local context, placing emphasis on human resources management. Recommendations for future implementation and sustainability include working with staff with technological literacy, safeguarding time allocation to the intervention activities and supervision, and ensuring flexibility when scheduling and conducting supervision meetings.

## Data Availability

The datasets used and/or analyzed during the current study are available from the corresponding author upon reasonable request.

## Abbreviations

LATIN-MH: Latin America Treatment Innovation Network in Mental Health
FHU: Family health unit
LMIC: Low and middle-income countries
RCT: Randomized control trial
NA: Nurse assistant
WHO: World Health Organization

## Footnotes

1 Within the Sao Paulo trial, nurse assistants and nurse technicians delivered the intervention. For the purpose of this article, they will be referred to as NAs.
